# Strategies for Promoting the Medical Device Industry in Korea: An Analytical Hierarchy Process Analysis

**DOI:** 10.3390/ijerph15122659

**Published:** 2018-11-27

**Authors:** Munjae Lee

**Affiliations:** Department of Medical Device Management and Research, SAIHST, Sungkyunkwan University, Seoul 06351, Korea; emunjae@skku.edu

**Keywords:** medical device industry, strategy, Korea, AHP

## Abstract

This study examines the medical device industry in the context of the Fourth Industrial Revolution and identifies the key strategies and general directions for promoting this industry through analytical hierarchy process (AHP). It is based on discussions of the key issues with specialists such as doctors, medical device companies, hospital staff, professors, and government agencies in the medical device industry. A total of 18 responses were obtained from the survey, and an analysis was conducted on the results. Among the medical device strategies identified by the surveyed specialists, clarifying the application of regulations (access strategy), increasing R&D investment for the technological development of medical devices (expansion strategy), and increasing support for global market expansion (infrastructure expansion strategy) were found to have the highest priority. Based on the above, we suggest key strategies and directions for promoting the medical device industry.

## 1. Introduction

The development of the Korean medical industry has increased the average lifespan and life expectancy of Korean citizens, while also improving their quality of life. Thus, Korea is investing in developing the health and medical industry to make it a strategic industry nationally [1]. In particular, the medical device industry has shown a high rate of growth, and this is expected to continue despite the global economic recession [2]. This growth is driven by the greater use of advanced medical devices aided by the development of IT skills. Indeed, the global medical device market is expected to grow to USD 446 billion by 2021, and the annual average growth rate after 2018 is estimated to be 5.8% [3,4].

The medical device market in Korea has grown at an annual average rate of 7.6% in the past five years, at least three times the rate of the global medical device market (2.2%). However, 82.4% of medical device manufacturers in Korea are companies with fewer than 20 employees, and 80.8% have annual sales below KRW 1 billion [2]. As small firms tend to lack the competencies to develop high-value products and have limited R&D investment, Korea’s market share and global competitiveness are low. Despite the business environment faced by such small medical device companies in Korea, however, the actual sales of medical devices in 2017 was KRW 5.82 trillion, a year-on-year increase of 3.9%, with exports increasing by 8.2%. Moreover, the number of workers engaged in the medical device industry was over 88,000, a year-on-year increase of 13.3%.

The medical device industry is a field of health and medical care that aims to improve the quality of life by manufacturing and selling medical devices, including products used to diagnose, treat, and prevent diseases, as well as to test, replace, and transform structures or functions. Medical devices are manufactured in small quantities and batches, with thousands of product types. Few items have a production volume exceeding 100,000 units. Hence, they are becoming more complicated and diverse with technological development, and the market is expanding because of the convergence of the medical device industry with information and communication technology (ICT) [3,5]. Indeed, this convergence has accelerated recently because of the onset of the Fourth Industrial Revolution, characterized by the integration of the digital and physical worlds, artificial intelligence (AI), and innovation in the field of biotechnology. Such convergence is also creating new industries by blurring the lines between cyberspace and the physical space [6].

In the Fourth Industrial Revolution, which has caused a paradigm shift in industries led by technologies such as AI, Internet of Things, big data, robotics, cloud computing, and 3D printing, the importance of industrial cooperation is growing, thereby creating new production methods [7]. As the lines between industries drawn by the Medical Services Act, Pharmaceutical Affairs Act, and Medical Devices Act become blurred, the technologies of the Fourth Industrial Revolution are expected to bring about changes in the health-based economy. Precision medicine, resulting from the convergence of ICT and healthcare, is likely to meet unsatisfied medical demand, while the increase in patients with chronic diseases is also shifting the medical paradigm [8]. In particular, the Korean medical device industry is commercializing customized medical devices, medical robots, and AI-based products by using 3D printing technology. Using advanced medical devices to reduce medical costs through prevention is thus serving as a new opportunity for the growth of the medical device industry, owing to the generalization of disease prevention and control [9,10].

Starting with the Fourth Industrial Revolution, in which automation and connectivity are maximized, new business models based on the intellectualization of things will be created; these may change the production and consumption of the products of all industries markedly. Hence, there are concerns that the rise in unemployment might worsen owing to the decrease in technical positions; however, it is also likely that new value-added jobs will emerge as society’s productivity and efficiency improve [11]. The medical device industry has a high ratio of value-added jobs. However, aside from the Medical Devices Act that enacts rules on medical devices, no legal system supports the development of this industry [12]. Moreover, although Korea has the world’s most highly qualified medical personnel, advanced technology, hospital information systems, and ICT skills, the development of its medical device industry is limited owing to insufficient R&D investment, a lack of global corporations, and an inadequate institutional base for the development of new industries. It is thus necessary to deal with the changes in the medical environment brought about by the Fourth Industrial Revolution by formulating key strategies for promoting the medical device industry. 

The paradigm of healthcare services is changing as the number of elderly people, chronic illnesses, and single-person households increases worldwide. The focus is shifting from treatment and diagnosis to precision and predictive and preventive care; moreover, with high medical expenses, interest in digital health care is increasing. The medical device industry is evolving rapidly, with key technologies leading the fourth industrial revolution. Personalized medical devices utilizing application products and 3D printing technology through fusion with ICT are expanding into a variety of products using advanced technologies such as medical robots, operating systems, artificial intelligence-based products, and virtual reality/enhancing reality. It is anticipated that products for the prevention and control of diseases will become universal and that products that combine various technologies such as information technology (IT), biotechnology (BT), and nanotechnology (NT) will create new medical demand and rapidly expand the market for medical devices. In addition, the medical device industry is experiencing an increasing opportunity for industrial growth due to changes in the industrial ecosystem caused by the emergence of destructive innovation technologies. By collecting data, analyzing it with ICT, and providing customized treatment, we are investing in precision medical care around the world because it can dramatically improve the quality of medical care and services and reduce astronomical medical costs. Therefore, an ICT base/competitiveness is being integrated with healthcare to promote research and development, and, by establishing a precision medical system, the expansion of precision medical care and the ICT–medical convergence are actively underway. In the changing medical paradigm leading to diagnosis, treatment, and prevention, the development of medical device products using medical Big Data extends the life of human beings and implements treatment methods that are less painful. In addition, the fourth Industrial Revolution combined with Big Data, ICT, and IT devices, will enable personalized treatment for individual life cycle healthcare management. Digital diagnosis, smart healthcare, and mobile devices used in individual health care are included in medical devices, and the importance of the medical device industry is further expanding. However, the current government regulations on medical devices have made it difficult to clearly apply new medical technologies to medical devices, and the entry of advanced devices into the market has been delayed. In addition, domestic medical device companies are experiencing difficulties in commercialization and export due to lack of licensing and manpower, so it seems urgent to secure specialist expertise [3].

The purpose of this study was to elucidate the major strategies and activation directions of the medical device industry through the analytic hierarchy process (AHP). In particular, the medical device industry is composed of more diverse stakeholders compared to other industries, and it is thought that deriving priorities through AHP analysis will help to forge the development direction of the medical device industry. This will contribute to the economic growth of Korea by providing a direction for the rapid development of the medical device industry as an industry for the future that creates new jobs.

## 2. Materials and Methods 

The AHP method selects the optimum alternative through a pairwise comparison of the importance of each attribute after hierarchically classifying multiple attributes [13]. AHP analysis was developed by Saaty and selects the important factors by prioritizing various elements. It sets the relative priority after determining and analyzing the problems to be solved through a structure comprising a number of hierarchies. AHP analysis is based on the judgment of the decision-maker about the relative importance of each criterion in terms of its contribution to the overall goal in addition to his or her preference for the alternatives related to each criterion; further, the judgment of the decision-maker must be based on objective data [14]. The relative importance of each bottom indicator is measured in terms of the top indicator, and pairwise comparison is then performed on a nine-point scale to derive the relative importance of the indicators. The values obtained by the pairwise comparison are used to calculate the consistency ratio (CR) of each component, thereby verifying the logical consistency of each judgment. These are considered to be consistent if the CR is 0.1 or lower; however, the pairwise comparison of the decision-maker must be reviewed again if it is above 0.1.

Through a survey, this study discussed the key issues with specialists such as doctors, medical device companies, hospital staff, professors, and government agencies in the medical device industry. The survey was conducted both online and offline, and all respondents were professionals who are engaged in the medical device industry. A total of 18 responses were obtained from the survey, and an analysis was conducted on the results. To identify and prioritize the key strategies and directions for promoting the medical device industry, this study used the categories of access, maintenance, expansion, and infrastructure expansion taken from Cho et al. [15]. Previous studies have identified high entry barriers in the industry, difficulties in long-term development investment, lack of infrastructure for ecosystem expansion, and lack of professional manpower discovery systems due to major issues and problems related to the macro-science industry ecosystem. The big science industry ecosystem is mostly dominated by business, and the need for long-term development investment, manpower, and advanced technology is similar to that of the medical device industry. Therefore, it seems reasonable to use the activation strategy derived from previous research [16].

However, the maintenance strategy was substituted with the competency enhancement strategy because the competencies of companies, research institutes, and government agencies in the medical device industry must be enhanced for the joint development of technology; there must also be organizational competency for the medical device industry to secure competitive advantage in the global market [17]. Thus, competency enhancement, access, expansion, and infrastructure expansion strategies were examined as the key strategies for the medical device industry (Table 1).

First, in terms of the competency enhancement strategy, Lee and Rim claimed that government support of software training is increasing, but the related education policy remains inadequate [13]. Given its current rate of growth, the medical device industry lacks specialists; thus, we suggest strategies for training staff in healthcare–ICT convergence as well as in medical device licensing and marketing for the long-term structural improvement of staff training and continuous growth of specialists in the medical device industry.

Second, as the access strategy, we adopted the policy conflict structure analysis model used in the broadcasting communications convergence promotion process of Shin and Kim [17]. To resolve conflicts among stakeholders, legislation must first be organized. As for demand, the government must ease regulations to allow firms to achieve external competitiveness and provide active support for the implementation of new technologies and industrialization. Moreover, there is also a need for policies to increase investment by improving R&D systems that promote the convergence of the broadcasting and communication industries. The medical device industry directly affects national health much more than other industries and thus has strict regulatory procedures; further, cooperation among stakeholders is important because medical devices have interdisciplinary convergence. To establish a network among stakeholders by resolving their conflicts owing to regulations on medical devices and develop their relationship into a cooperative one, we suggest strategies for clarifying the application of regulations related to medical devices, building an ecosystem for the convergence of the medical device industry, discovering new companies, and supporting development costs [18].

Third, as the expansion strategy, we used the fact that most projects are led by the government owing to the nature of big science, as shown by Cho et al.; however, at the ground level, projects are conducted on the basis of cooperative relations with small and medium-sized enterprises (SMEs) that have excellent technological skills [15]. In Korea, specialized SMEs are leading the medical device market for low-priced products, whereas a few conglomerates lead the high-priced, advanced value-added products market. Therefore, to develop the medical device industry by promoting partnerships between SMEs with core technologies and conglomerates with abundant capital to develop and utilize technology, we suggest strategies for promoting partnerships in medical device businesses, increasing R&D investment for the technological development of medical devices, supporting convergence in the development of healthcare IT, and promoting information sharing in local and global medical device markets.

Finally, as the infrastructure expansion strategy, we used the infrastructure expansion and industrial environment aspects of the study of plans to develop the Korean biotechnology industry by Ku et al. [19]. Korean medical device firms have lower awareness than global corporations overseas; thus, governmental efforts are required for foreign market expansion. Hence, we suggest strategies for increasing support for global market expansion and promoting startups in the medical device industry.

**Figure 1:**
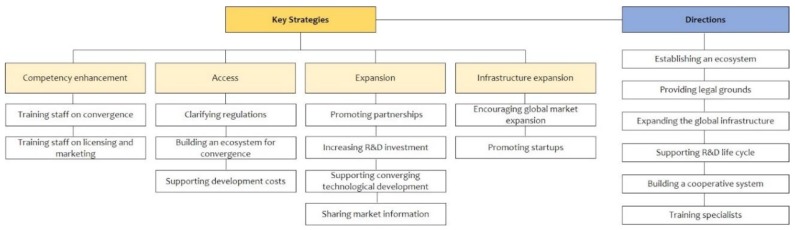
Research framework.

Next, we suggest six elements based on previous studies to set the directions for promoting the medical device industry (Table 2). We took elements such as establishing a key staff training system, building an ecosystem for startups, and enhancing comprehensive R&D competencies from Hyun and Kim [20], as the models in their study focus on promoting the convergence of the Fourth Industrial Revolution and cultural and creative industries, as well as creating jobs. The changes in the industrial structure brought about by the Fourth Industrial Revolution will also affect staff competencies. Therefore, to enhance competencies and expand technologies in the medical device industry, we suggest strategies for training specialists and supporting technology and businesses for the entire R&D life cycle [20]. Lee and Hwang, in their study of ways to increase exports, suggested a strategy for expanding infrastructure, namely, that companies must participate in international expositions and use global buyers to expand into overseas markets [21]. Lee claimed that various stakeholders must build an industrial ecosystem to promote IT convergence [22]. This suggested strategies such as the establishment of an industrial ecosystem by medical device industry stakeholders with different stances, the management of conflicts between the private and the public sector, and the establishment of a cooperative system through the collaboration of the government and related companies. Figure 1 illustrates the evaluation items and hierarchical structure [23].

## 3. Results

The AHP analysis in this study was conducted using the AHP-specific solution, DRESS (1.7.00, CHOISH Software DRESS, Korea). DRESS calculates the relative importance of the attributes by using the eigenvector method in the pairwise comparison among the attributes of each hierarchy. Moreover, it calculates the relative importance of each respondent and comprehensive relative importance by using their means. The CR is supposed to review the logical consistency of the evaluator, which must be less than 0.1 for it to be considered reliable. This study conducted online and offline surveys with 18 specialists in the medical device industry, and the CR of the AHP results was less than 0.1, thereby ensuring reliable results. Most of the surveyed individuals worked in medical device companies, followed by government employees, doctors, and hospital personnel. Altogether, 61.2% of the professionals had worked in the related field for over 10 years, and 55.6% were over 40 years old (Table 3).

By analyzing the importance of the key strategies for promoting the medical device industry, it was found that the strategy with the highest importance was clarifying the application of regulations (0.14), followed by increasing R&D investment for the technological development of medical devices (0.12), increasing support for global market expansion (0.12), supporting convergence in the development of healthcare IT (0.10), promoting information sharing by local and global medical device markets (0.10), training staff in medical device licensing and marketing (0.09), promoting startups in medical devices (0.09), building an ecosystem for the convergence of the medical device industry (0.07), discovering new companies and supporting development costs (0.07), promoting partnerships among medical device businesses (0.06), and training staff in healthcare–ICT convergence (0.05). Hence, in terms of the strategies for promoting the medical device industry, the surveyed specialists think that it is necessary to clearly apply regulations (access strategy), increase R&D investment for technological development (expansion strategy), and increase government support for global market expansion (infrastructure expansion strategy). Table 4 presents the survey results.

By analyzing the importance of the directions for promoting the medical device industry, the strategy with the highest importance was found to be training specialists (0.26), followed by expanding infrastructure for the global expansion of medical devices (0.19), establishing legal grounds to expand market access (0.17), supporting technology and businesses during the entire R&D life cycle (0.15), managing conflicts between the private and the public sector and building a cooperative system (0.15), and establishing the ecosystem for stakeholders of medical devices (0.08). Hence, the surveyed specialists think that it is important to develop technologies for medical devices, along with training specialists, expanding the infrastructure to grow the medical device market, expanding market access by clarifying the application of regulations, and establishing legal grounds for that. Table 5 presents the results of the analysis.

## 4. Discussion

In terms of the priority of the medical device strategies identified by the surveyed specialists, clarifying the application of regulations (access strategy), increasing R&D investment for the technological development of medical devices (expansion strategy), and increasing support for global market expansion (infrastructure expansion strategy) were found to have high priority. The strategy for promoting the medical device industry with the highest importance was training specialists in the medical device industry, followed by expanding infrastructure for global expansion of medical devices, and establishing legal grounds to expand market access. We infer from this that the most urgent problem in the medical device industry is to clarify regulations and expand market accessibility. In addition, a larger R&D investment is needed to develop medical device technology and to train specialists to use it. Support for entry into foreign markets is provided through the infrastructure of the global market. Based on the above, we thus suggest key strategies and directions for promoting the medical device industry.

First, it is necessary to establish clear legal grounds for access to the medical device market by applying regulations. Medical devices have direct and indirect effects on national health and on securing the right to health; thus, they require regulations such as government licensing. Moreover, they are directly linked to national health, and the market entry barrier for medical devices is higher than in other industries [24]. The government manages medical devices by regulating all functions, such as production, manufacturing, clinical testing, distribution, and sales. Before they enter the market, medical devices must undergo licensing, the verification of existing technology, new health technology assessment, and health insurance payment, all of which can take up to 520 days. Although safety and effectiveness must take precedence in the case of medical devices, there is also criticism over the double regulation, namely, having to receive assessment on the existing technology as well as on the new health technology after licensing. These strict regulatory procedures prevent a flexible response to the rapid industry changes. Regulations are thus one of the important policy means that affect the innovative activities required by firms or industries to enhance competitiveness; however, in new industries with rapid technological development, such as the medical device industry, regulations sometimes hinder innovation because they fail to keep pace with technological development [25].

It is difficult to clearly determine the possibility of market entry for medical devices that are being developed, owing to complicated regulatory procedures [26]. Medical devices are developed by combining the regulation information for each step; however, information is disclosed separately by each of the regulatory agencies, and thus multiple agencies must be contacted individually. For companies that need information throughout the life cycle, from R&D to market entry, this method has its limitations for obtaining integrated information because the data are scattered. To solve this problem, the Ministry of Health and Welfare, Ministry of Food and Drug Safety, National Evidence-based Healthcare Collaborating Agency, Health Insurance Review and Assessment Service, and Korea Health Industry Development Institute provide medical device businesses with integrated consultation during the entire life cycle on the regulatory systems and procedures, as well as serve as a comprehensive support center for medical devices. Moreover, efforts are made to induce prompt market entry by simplifying the procedures for assessing new healthcare technology. Assessing and licensing new healthcare technology are carried out simultaneously when the purpose of health technology is similar or the same, whereas separate procedures for reducing this period must be implemented for medical devices not subject to integrated assessment. Thus, both the assessment and the deliberation of insurance registration will be carried out if cost-effectiveness data are submitted to the Health Insurance Review and Assessment Service under this new health technology assessment, even if the company is not subject to integrated assessment.

Recently, with the onset of the Fourth Industrial Revolution, various product lines using advanced technology have been developed rapidly; however, their market entry has been delayed owing to a lack of research findings proving their safety and effectiveness because of their short development history. To solve this problem, these organizations are establishing a new classification system for newly developed medical devices and creating guidelines to promptly license them once developed. Furthermore, it is necessary to promote market access and the entry of innovative medical devices by ensuring regulations are flexible. This can be done by understanding how medical devices that are convenient for medical staff and increase productivity can enter the market without going through a new health technology assessment [27,28].

Second, training specialists in medical devices and technological development and raising R&D investment must be carried out simultaneously. The medical device industry is a capital- and technology-intensive industry monopolized by a few multinational companies with high technology and marketing skills. Products from global companies dominate the Korean market, and thus it is difficult to break away from conservative product preferences. In these circumstances, the utilization rate of Korean medical devices is low owing to their substandard performance, reliability, and clinical evidence compared with foreign products. Korea’s medical device technology is similar to that of China, which is a latecomer in the field, and generally falls short of that in advanced countries. It is therefore necessary to reinforce competitiveness by developing competencies. However, the R&D expenditure of all Korean medical device companies is less than 25% of that of a single foreign company. This is because medical device manufacturers in Korea are mostly SMEs that have limited budgets for investing in improving product performance or securing reliability.

R&D investment in the medical device industry affects not only new product development but also quality and process improvements [29]. The manufacturing capacity is closely related to business performance, and thus it is important to secure product reliability through constant quality improvement by increasing R&D investment; this will enhance business performance [5]. The government’s R&D investment helps constantly improve firm competitiveness along with national competitiveness [30,31]. Moreover, the government’s R&D investment in SMEs leads to rising R&D investment by the firms. Therefore, the government can create jobs and vitalize the economy by increasing the R&D budget and fostering development by SMEs [31,32]. The government’s R&D investment in medical devices in 2016 was KRW 366.5 billion, 14.6% of the R&D investment in health and medical treatment. The average annual increase is 11.3%, indicating that R&D investment is constantly rising compared with that in other fields [33].

The medical device industry is changing rapidly with the expansion of big data and AI as well as the convergence in the development of healthcare ICT. Firms are trying to develop medical devices for prevention, diagnosis, treatment, rehabilitation, and management by supporting converging technological developments in healthcare, such as intelligent body-implanted medical devices and AI-based robot medical tools. Moreover, they are providing healthcare–ICT convergence solutions to improve the current medical environment and achieve new clinical breakthroughs. However, in the current system, the Ministry of Science and ICT supports basic and applied research, the Korea Health Industry Development Institute supports the manufacturing of medical devices, and the Ministry of Health and Welfare supports clinical tests and commercialization. Because the research findings are spread among research institutes, firms, and hospitals, they are not used in hospitals. This scattered R&D support system across various departments deteriorates investment efficiency and restricts the effectiveness of measures against environmental change. Thus, an integrated R&D project covering all departments needs to be carried out to establish a support system for developing the entire life cycle of medical device technologies. In some cases, firms fail to enter the market because they develop products without considering licensing and insurance registration. Hence, by supporting licensing technology and training for the entire life cycle and establishing plans for local market entry and new global market expansion, it will be possible to manufacture medical devices quickly and accelerate market entry. This integrated R&D project will also resolve the issue of the inefficiency caused by redundant R&D investment in medical devices by different departments.

Innovative products are developed to promote the competitiveness of the medical device industry, and greater R&D support and investment enhance productivity; however, there must exist organizations with specialized knowledge and expertise to improve performance [34]. Korean medical device companies lack intellectual capital because outstanding employees avoid working at small firms. The government plans to establish a professional graduate school focused on medical devices to foster staff with specialized knowledge and expertise in medical devices. Although specialized graduate schools do nurture specialists with multidisciplinary knowledge about the characteristics of the medical device industry, there is a growing need for a degree system with a balance of academia, industry, and employment to ensure the effective training of high-quality staff. Producing staff in the medical device industry through such professional graduate schools will lead to greater expertise in Korean companies and the creation of jobs [35,36].

Third, the government must establish a global infrastructure for medical devices by increasing support for the global expansion of Korean medical device companies. In Korea, medical device companies compete with one another in a small domestic market; thus, new value creation is desperately needed through the expansion of exports. To this end, it is first necessary to promote the reliability of Korean medical devices through performance testing and product improvement. The competitiveness of products and services is fundamental for successful overseas expansion. However, it is important for SMEs to obtain information about environmental factors before competing with multinational companies [37]. Furthermore, to create higher value through overseas market expansion, it is necessary to deal with global licensing procedures that are stricter than those in Korea. To this end, the Korean government provides consulting related to the acquisition of international certification to meet international standards through the Medical Device Information & Technology Assistance Center. Recently, Korea joined the International Medical Device Regulators Forum (IMDRF) as an official member. All IMDRF members apply the Medical Device Single Audit Program (MDSAP) that acknowledges some or all good manufacturing practices (GMP) in medical devices. The MDSAP will shorten the licensing period and thus aid the export of Korean medical devices.

Opportunities are growing in the medical device industry in emerging countries. However, Korean companies are facing barriers to market entry, owing to a lack of understanding of the different industrial structures and markets of each country and difficulty in obtaining information about global medical institutions and European buyers. Thus, a database of overseas medical device markets in each country is to be established. Further, market expansion data on global medical devices, such as the market size and trading status in each country as well as information on major exporting countries, are to be provided through the Unique Device Identification system in operation.

Recently, it has become necessary for companies to enter overseas markets. Companies expanding overseas transfer their own resources to overseas subsidiaries to compete with local companies [38]. However, Korean medical device companies with insufficient funds face difficulties in expanding overseas through local production. It is therefore essential to establish local corporations or branches to develop overseas markets and conduct normal sales activities despite the burden of additional operating costs. Thus, the government is about to set up support measures for Korean companies to establish their bases overseas to help them increase exports and expand outside Korea. The government will bear the consulting expenses for law, accounting, and tax affairs to establish an overseas corporation, thereby helping a firm overcome the difficulty of entering the global market. The government also supports the establishment of a sales network by covering expenses for participating in exhibitions and conferences and holding seminars. In particular, China has high market entry barriers because public hospitals must ensure that 70% of the products they use are Chinese to promote the domestic market. Further, the process of purchasing imported goods is complicated. To resolve this inequality issue, Korean medical companies are undergoing localization in China. They must also actively aim for international harmonization [39].

## 5. Conclusions

With the aging of society, the paradigm of battling diseases is shifting from treatment to diagnosis and prevention. The fields of medicine, medical devices, and services are showing constant growth, as demand for health and medical care rises. In particular, the medical device industry is anticipating the creation of high-quality jobs [40]. However, Korean medical device companies are small and have inadequate staff competencies and product durability; thus, doctors distrust Korean medical devices, and tertiary hospitals tend to avoid domestic products. This is reflected in the low market share of domestic products in the Korean medical device market. However, low profits prevent companies from growing, thereby resulting in a vicious cycle. It is thus necessary to overcome these limitations by establishing strategies for promoting the Korean medical device industry given environmental changes such as increased demand for medical devices owing to higher expenditure and the expansion of industrial size and scope with the emergence of the Fourth Industrial Revolution [3,10].

This study identifies the key strategies and directions for promoting the medical device industry through an AHP analysis, thus contributing to the continuous development of this industry as a national growth engine. The AHP analysis of surveyed specialists of the medical device industry derived a number of strategies. The strategies with the highest priority were clarifying the application of regulations (access strategy), increasing R&D investment for the technological development of medical devices (expansion strategy), and increasing support for global market expansion (infrastructure expansion strategy). After analyzing the priority directions for promoting the medical device industry, this study finds that the strategies with the highest priority were training specialists in the medical device industry followed by expanding infrastructure for the global expansion of medical devices and establishing legal grounds to increase market access [41].

The medical device industry is a typical field of the Fourth Industrial Revolution in that it is developing rapidly by applying advanced technologies such as AI, big data, and robotics. The government has been supporting the medical device industry by increasing R&D investment and shortening the period in which regulations are in place. However, regulations fail to keep pace with the rapid technological development of medical devices. Thus, President Moon Jae-in announced a plan for the regulatory innovation and industrial development of medical devices and decided to implement a comprehensive negative regulation on medical devices, with little concern about safety and permitting market entry with only ex post facto evaluation. In vitro diagnostic medical devices became the first target of this negative regulation and were excluded from new health technology assessment. Regulations not only reduce national concern about the harmful effects of technology innovation, but also hinder technology innovation. Therefore, regulations must perform a leading role in creating an environment that induces innovation and investment [42]. To develop the medical device industry, it is necessary to actively reform regulations, train staff by reinforcing R&D investment, and facilitate overseas market expansion. This type of growth of the medical device industry would have a positive effect on the Korean business structure and on job creation.

This study may have some limitations. The review of preceding studies was limited, and the AHP analysis results were not compared with an occupational group. It is difficult for the medical device industry to reach consensus on the opinions of government and medical device companies. Future research will need to prioritize a comparison between government agency and medical device company opinions. Nevertheless, this study has constructed a strategy based on the experiences of similar industries under the condition of limited prior research on the domestic medical device industry. It can thus contribute greatly in terms of deriving strategic priorities for the medical device industry.

## Figures and Tables

**Table 1:** Key strategies for promoting the medical device industry.

Strategy	Detailed Strategy	Selection Criteria
Competency enhancement	Training staff in healthcare—information and communication technology (ICT) convergence	Training staff
	Training staff in medical device licensing and marketing	Training staff
Access	Clarifying the application of regulations	Resolving stakeholder conflict through legal reform
	Building an ecosystem for convergence in the medical device industry	Encouraging stakeholder cooperation
	Discovering new companies and supporting development costs	Strengthening government support for new technology
Expansion	Promoting partnerships among medical device businesses	Promoting partnerships in the medical device industry
	Increasing R&D investment for the technological development of medical devices	Investing in the technological development of medical devices
	Supporting convergence in the development of healthcare information technology	Developing medical device core technology
	Promoting information sharing in local and global medical device markets	Sharing information through partnerships
Infrastructure expansion	Increasing support for global market expansion	Providing government support to improve awareness
	Promoting startups in the medical devices market	Offering startup support to expand the global market

**Table 2:** Directions for promoting the medical device industry.

Evaluation Item	Selection Criteria
Establishing an ecosystem for the stakeholders of medical devices	Establishing an ecosystem for developing the medical device industry
Establishing legal grounds to expand market access	Establishing a legal basis for fast market entry
Expanding infrastructure for the global expansion of medical devices	Expanding the overseas market for domestic medical device companies
Supporting technology and businesses during the entire R&D life cycle	Expanding medical device industry technology
Managing conflicts between the private and the public sector and building a cooperative system	Encouraging governments and companies to collaborate to benefit the medical device industry
Training specialists in the medical device industry	Training staff on systematic construction

**Table 3:** Summary statistics.

Characteristics		Frequency	Ratio (%)
Sex	Male	11	61.1
	Female	7	38.9
Age	20s	2	11.1
	30s	6	33.3
	40s	7	38.9
	50s	3	16.7
Work experience in the related field	Under 5 years	3	16.7
	5–10 years	4	22.2
	10–20 years	10	55.6
	Over 20 years	1	5.6
Occupation	Doctor	2	11.2
	Business	8	44.8
	Hospital worker	2	11.2
	Professor	2	11.2
	Civil servant	3	16.7
	Other	1	5.6

**Table 4:** Analyzing the key strategies for promoting the medical device industry.

Strategy	Factor		
	Importance	Priority	CR
Competency enhancement			0.01
Training staff on convergence Training staff on licensing and marketing	0.05	11	
	0.09	6	
Access			
Clarifying regulations Building an ecosystem for convergence supporting development costs	0.14	1	
	0.07	8	
	0.07	8	
Expansion			
Promoting partnerships Increasing R&D investment Supporting converging technological development Sharing market information	0.06	10	
	0.12	2	
	0.10	4	
	0.10	4	
Infrastructure expansion			
Encouraging global market expansion Promoting startups	0.12	2	
	0.09	6	

**Table 5:** Analyzing the directions for promoting the medical device industry.

Goal	Factor		
	Importance	Priority	CR
Establishing an ecosystem Providing legal grounds Expanding the global infrastructure Supporting the R&D life cycle Building a cooperative system Training specialists	0.08	6	
	0.17	3	
	0.19	2	
	0.15	4	
	0.15	4	
	0.26	1	
